# A review of data sharing statements in observational studies published in the BMJ: A cross-sectional study

**DOI:** 10.12688/f1000research.12673.2

**Published:** 2017-11-06

**Authors:** Laura McDonald, Anna Schultze, Alex Simpson, Sophie Graham, Radek Wasiak, Sreeram V. Ramagopalan

**Affiliations:** 1 Centre for Observational Research and Data Sciences, Bristol-Myers Squibb, Uxbridge, UB8 1DH, UK; 2 Real-World Evidence, Evidera, London, W6 8DL, UK

**Keywords:** Data-sharing, real-world data, observational

## Abstract

In order to understand the current state of data sharing in observational research studies, we reviewed data sharing statements of observational studies published in a general medical journal, the British Medical Journal. We found that the majority (63%) of observational studies published between 2015 and 2017 included a statement that implied that data used in the study could not be shared. If the findings of our exploratory study are confirmed, room for improvement in the sharing of real-world or observational research data exists.

## Introduction

Over the recent years, a number of articles and movements have called for the sharing of clinical trial data ^1– 3^. However the access to and sharing of real-world/observational data receives little and arguably insufficient attention. In this study we sought to assess the current state of data sharing in published observational studies in a general medical journal, namely the British Medical Journal (BMJ). The BMJ was chosen as the journal does not enforce a commitment of data sharing for observational studies ^4^, but all research articles are required to contain a data sharing statement ^5^.

## Methods

All observational research articles published in the BMJ between 1 ^st^ January 2015 and 31 ^st^ August 2017 were investigated. These dates were chosen as it provides over 100 articles for analysis and recent data post-dating some of the articles regarding clinical trial data sharing ^2^. Assuming a proportion of articles not being shared of between 20–40%, 100 articles would enable a precision of 7.8 to 9.6%. Observational research articles were defined as cohort, case-control and cross-sectional studies, as well as case series. Meta-analyses, systematic reviews, randomised controlled trials and genetic/Mendelian randomisation studies were excluded. The data sharing statements of these studies were reviewed. If the statement written was “no additional data available” ^5^ or that data was not publicly available, the study data was classed as not shared. Statements alluding to data being available from the corresponding author, or a referral to access policies of the data source the study data was classed as shared. Where statements alluded to code or technical appendix being available, but no reference to data specifically being available, the study data was classed as not shared.

## Results

Two hundred and thirty seven observational studies were included. A review of the data sharing statements of these studies revealed that 149 (63%) studies did not share data.

Raw data showing the studies identified from the BMJ and whether the data sharing statements indicated data was not/were available Click here for additional data file. Copyright: © 2017 McDonald L et al. 2017 Data associated with the article are available under the terms of the Creative Commons Zero "No rights reserved" data waiver (CC0 1.0 Public domain dedication).

## Conclusions

In our review of the data sharing statements of observational studies published in the BMJ, we identified that the majority of studies did not share data. Whilst there are likely many reasons for this, including patient confidentiality concerns and the access possibilities of the data source, the lack of data sharing in observational research is a potential cause for concern. The key limitation of our study was the scope of the data. We only reviewed the data sharing statements of one medical journal and therefore the generalisability of our results is unclear. The data sharing policy of the BMJ is relatively general (sharing is encouraged, but specific requirements are not communicated). Follow on work should assess how the robustness of a journal’s data sharing policy could influence the rate of data sharing. Our analysis should be seen as exploratory rather than definitive. Further studies are needed, in greater depth, to confirm or refute our findings. If consistent findings are seen, the lack of sharing of observational research data is an area that warrants further attention.

## Data availability

The data referenced by this article are under copyright with the following copyright statement: Copyright: © 2017 McDonald L et al.

Data associated with the article are available under the terms of the Creative Commons Zero "No rights reserved" data waiver (CC0 1.0 Public domain dedication). 

Dataset 1: Raw data showing the studies identified from the BMJ and whether the data sharing statements indicated data was not/were available. doi, 10.5256/f1000research.12673.d177871 ^6^

## References

[1] Goldacre B Lane S Mahtani KR: Pharmaceutical companies’ policies on access to trial data, results, and methods: audit study. *BMJ.* 2017;358:j3334. 10.1136/bmj.j3334 28747301

[2] Krumholz HM Peterson ED: Open Access to Clinical Trials Data. *JAMA.* 2014;312(10):1002–1003. 10.1001/jama.2014.9647 25203080

[3] AllTrials: All Trials Registered. All Results Reported. *AllTrials.* Reference Source

[4] Research | The BMJ. (Accessed: 2nd September 2017). Reference Source

[5] Groves T: Sharing the raw data from medical research [corrected]. *BMJ.* 2009;338:b1252. 10.1136/bmj.b1252 19321550

[6] McDonald L Schultze A Simpson A: Dataset 1 in: A review of data sharing statements in observational studies published in the BMJ: A cross-sectional study. *F1000Research.* 2017 Data Source 10.12688/f1000research.12673.1 PMC5676190 29167735

