# Supplementary material for: A review of data sharing statements in observational studies published in the BMJ: A cross-sectional study
Source: F1000Res. 2017 Nov 6;6:1708. Originally published 2017 Sep 19. [Version 2] doi: 10.12688/f1000research.12673.2 (PMC5676190; doi:10.12688/f1000research.12673.2)
Supplement: Peer review history

# Peer review: Referee response for version 2

**DOI:** 10.5256/f1000research.14218.r27649

**Published:** 2017-11-07

**Authors:** Robin E Champieux

**Affiliations:** 1 OHSU Library, Ontology Development Group, Oregon Health & Science University, Portland, OR, USA

The authors sufficiently addressed my original concerns.  Overall, this study offers a valuable jumping off point for further investigations in this area.

I have read this submission. I believe that I have an appropriate level of expertise to confirm that it is of an acceptable scientific standard.

# Peer review: Referee response for version 1

**DOI:** 10.5256/f1000research.13724.r26439

**Published:** 2017-11-02

**Authors:** Robin E Champieux

**Affiliations:** 1 OHSU Library, Ontology Development Group, Oregon Health & Science University, Portland, OR, USA

This exploratory analysis contributes to the literature on biomedical data sharing practices, and demonstrates the importance of the further understanding of data sharing policies, practices, and barriers for domain and type specific data sets, such as those associated with observational studies.

 While it does not impact the soundness of this short report, I would recommend that the authors expand on their reasoning for choosing BMJ for their analysis.  Specifically, while BMJ requires data sharing statements, its data sharing policy is relatively general (sharing is encouraged, but specific requirements are not communicated).  It would be helpful for the authors to discuss (potentially as a follow-up analyses), how the robustness of a journal's data sharing policy could influence the rate of data sharing.

 Regarding the sampling methodology, the authors write, "All observational research articles published in the BMJ between 1st January 2015 and 31st August 2017 were investigated. These dates were chosen as it provides a reasonable sample size for analysis and recent data post-dating some of the articles regarding clinical trial data sharing."  In my view, this statement is insufficient.  The authors should provide more detailed and quantitative information about the strength and the nature of the sample.

 Finally, the authors write "A review of the data sharing statements of these studies revealed that 149 (63%) studies had a statement implying that the data underlying the study could not be share".  However the methodology does not indicate if the data "could" be shared, only if it was shared.  I would urge the authors to revise this statement be be more precise.

I have read this submission. I believe that I have an appropriate level of expertise to confirm that it is of an acceptable scientific standard, however I have significant reservations, as outlined above.

# Response

**Published:** 2017-11-02

**Authors:** Sreeram Ramagopalan

We thank the reviewer for their comments. We focussed on the BMJ because of the journal being a key backer of the all trials initiative but we agree with the reviewers point and have updated the discussion.

 We have updated the methods to include a precision calculation.

 We corrected the results sentence.

# Peer review: Referee response for version 1

**DOI:** 10.5256/f1000research.13724.r26085

**Published:** 2017-09-26

**Authors:** Laurie Tomlinson

**Affiliations:** 1 Faculty of Epidemiology and Population Health, London School of Hygiene & Tropical Medicine, London, UK

This is an interesting study that draws attention to an important area - open access to the data underpinning research papers allowing assessment of reproducibility. It is of course limited by it's focus on one journal as data-sharing policies vary between journals. In addition, requested statements by journals often become formulaic as the tendency is to copy what other papers have said previously. Some journals have tried new approaches in an effort to encourage data-sharing such as the NEJM 'Sprint Challenge'.

 However, the major limitation is a lack of further data on why data was not shared. Some providers such as the UK primary care data repository CPRD do not allow data-sharing, even if the authors of articles are keen proponents of open data-access. More in depth work will be required to understand the true barriers and way ahead for data-sharing.

I have read this submission. I believe that I have an appropriate level of expertise to confirm that it is of an acceptable scientific standard.
